# Supplementary material for: Marine-Derived N-Terminal Mitochondrial-Targeting Sequences Exhibit Antimicrobial and Anticancer Activities
Source: Int J Mol Sci. 2025 Sep 3;26(17):8546. doi: 10.3390/ijms26178546 (PMC12429151; doi:10.3390/ijms26178546)
Supplement: Supplementary file 1 [file ijms-26-08546-s001.zip › ijms-3764121-supplementary.pdf]

**Supplementary Materials**

**Figure S1.** Analysis of helix-wheel diagrams and secondary structures of selected MTS

| No. | Organism | Gene | Structure | |
|---|---|---|---|---|
| | | | Helix-wheel | 2nd |
| 1 | Crab<br>(P. trituberculatus) | E2C01 | 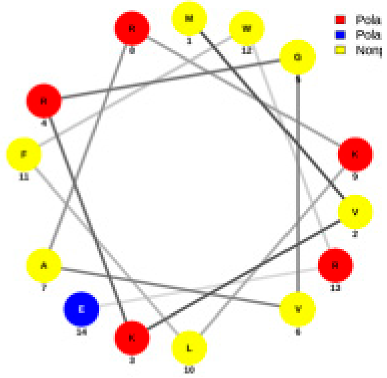 | 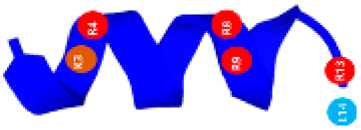 |
| 2 | | CLPP | 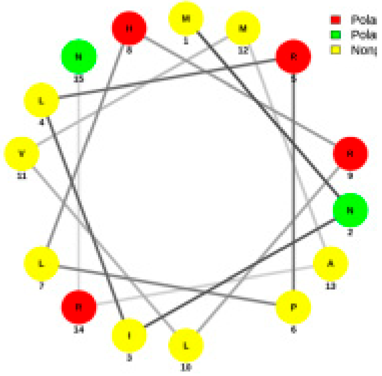 | 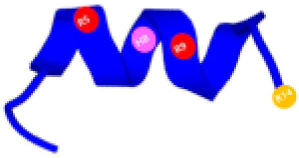 |
| 3 | | Tmlhe | 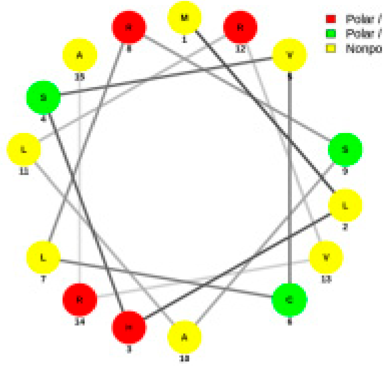 | 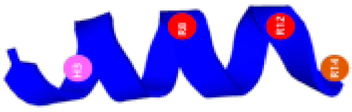 |
| 4 | | ES1 | 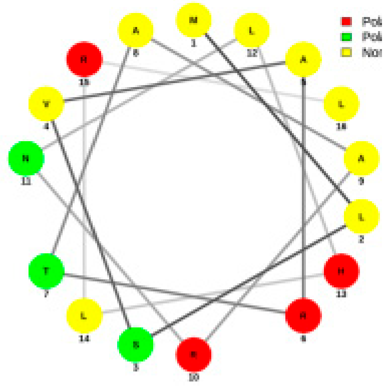 | 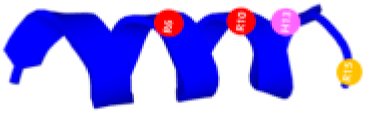 |

| | | | | |
|---|---|---|---|---|
| 5 | | TK2 | 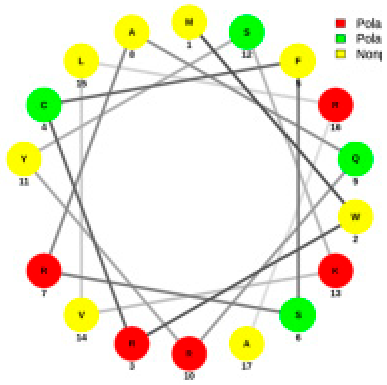 | 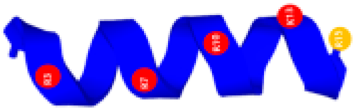 |
| 6 | | Mmaa | 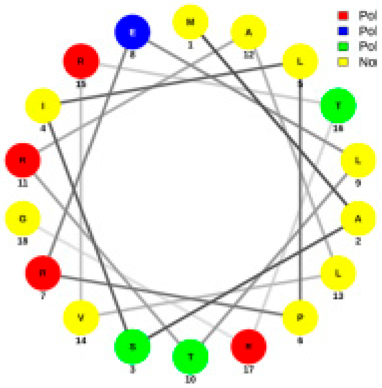 | 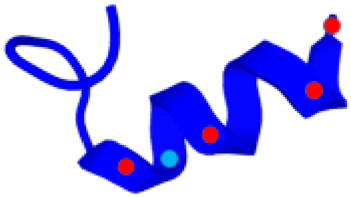 |
| 7 | | Echdc3 | 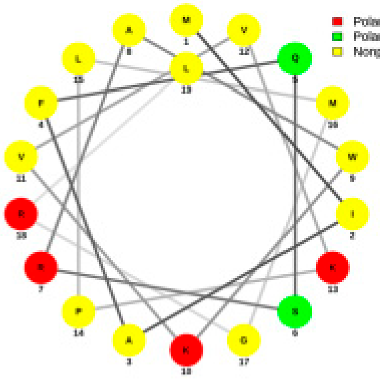 | 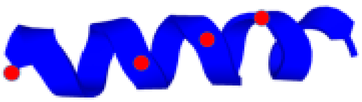 |
| 8 | | COX42 | 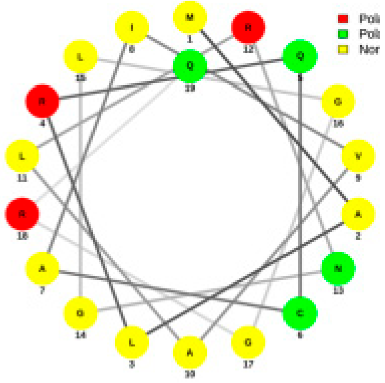 | 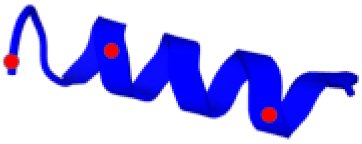 |

| | | | | |
|---|---|---|---|---|
| 9 | | Atp5d | 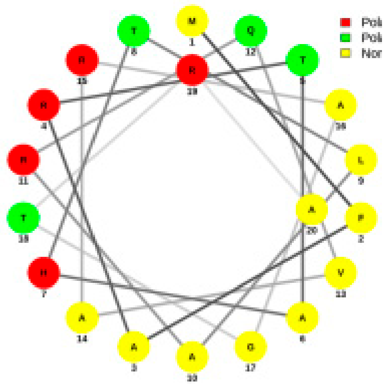 | 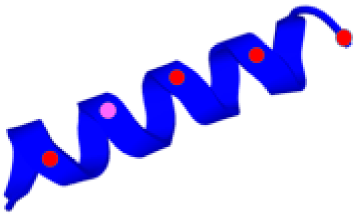 |
| 10 | | MCCC1 | 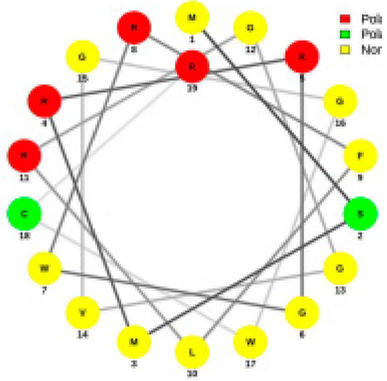 | 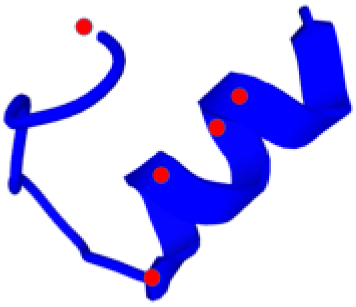 |
| 11 | | Auh | 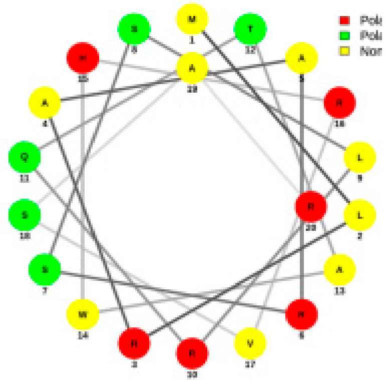 | 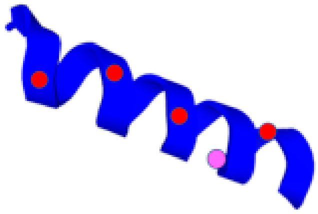 |
| 12 | | PMPCA | 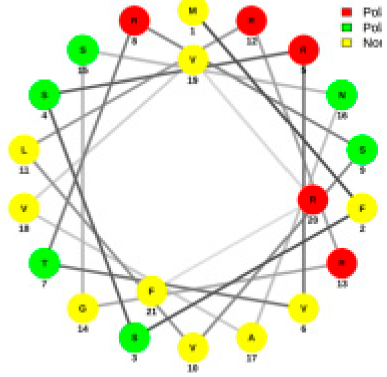 | 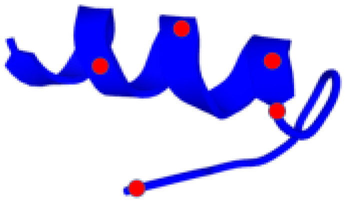 |

| | | | | |
|---|---|---|---|---|
| 13 | | MRPL50 | 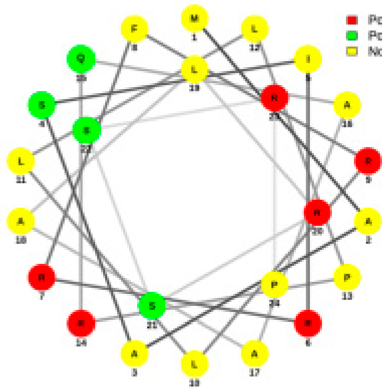 | 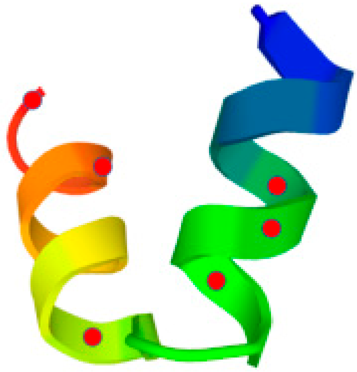 |
| 14 | | Mrpl4 | 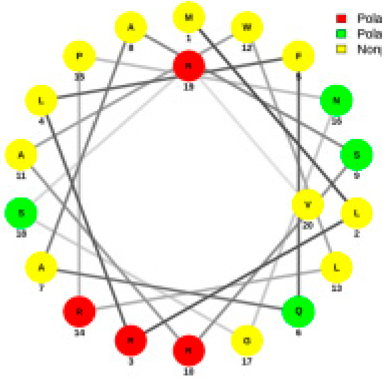 | 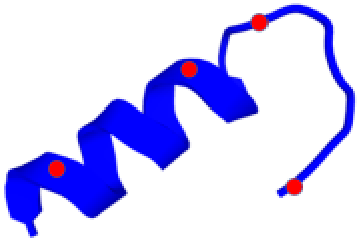 |
| 15 | | Acot9 | 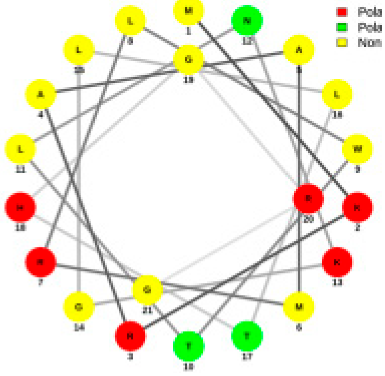 | 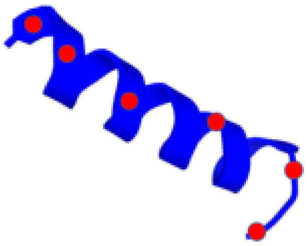 |
| 16 | Herring<br>(D. clupeoides) | DBT | 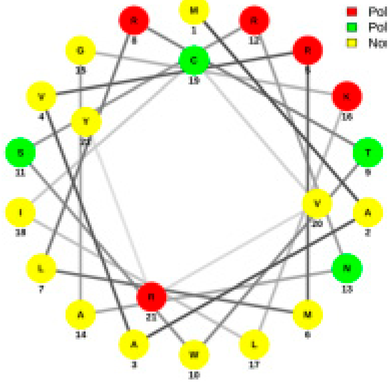 | 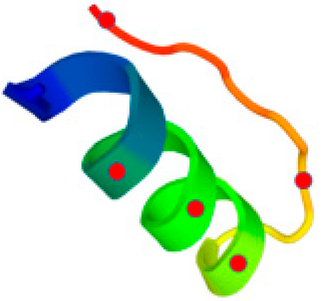 |

| | | | | |
|---|---|---|---|---|
| 17 | Shrimp<br>(P. chinensis | SOD | 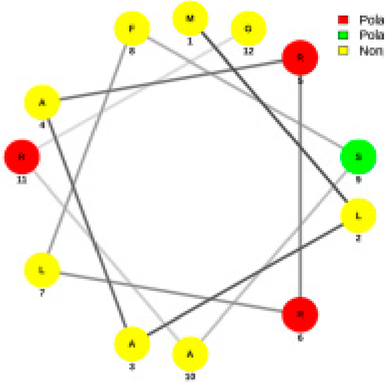 | 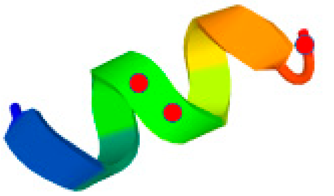 |
| 18 | Oyster<br>(C. gigas) | Sdi1 | 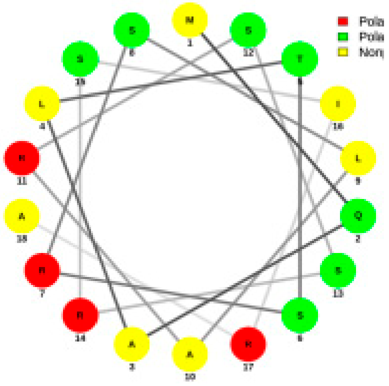 | 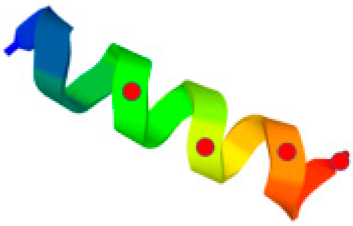 |
| 19 | | MIMP1 | 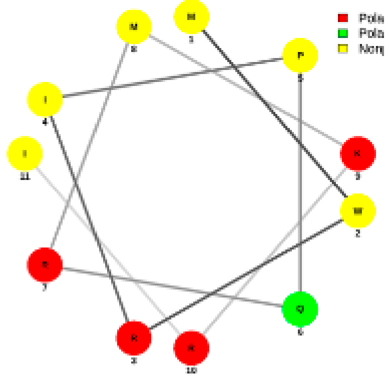 | 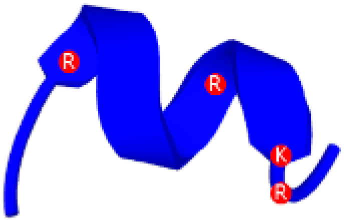 |
| 20 | Brown algae<br>(E. siliculosus) | TIM44 | 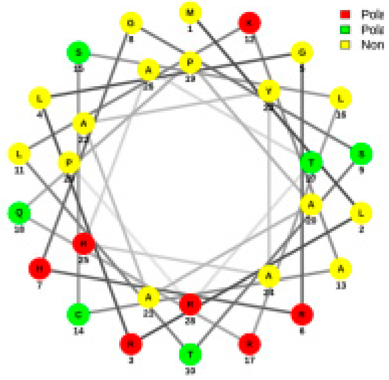 | 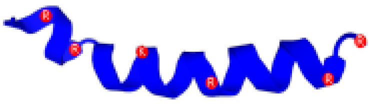 |

| | | | | |
|---|---|---|---|---|
| 21 | Whale (T. truncatus) | TIM50 | 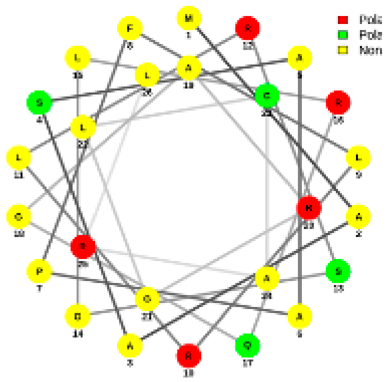 | 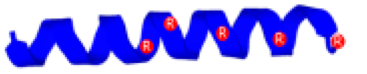 |
| 22 | Whale (P. catodon) | Tim29 | 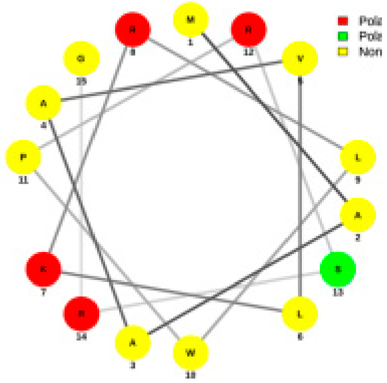 | 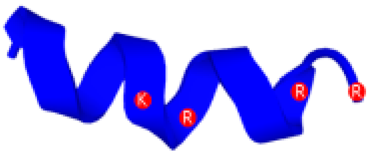 |
| 23 | Whale (B. musculus) | Tim29 | 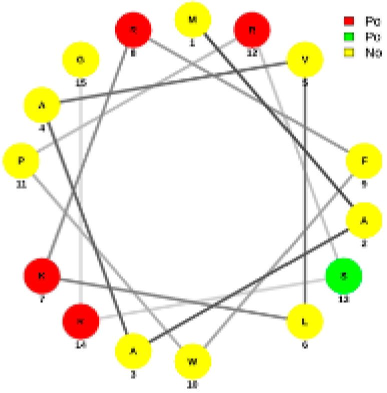 | 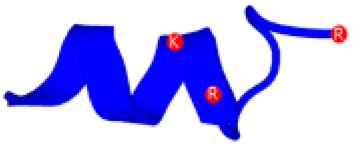 |
| 24 | Salmon (O. nerka) | AIF1 | 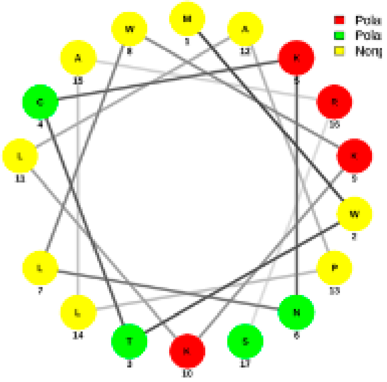 | 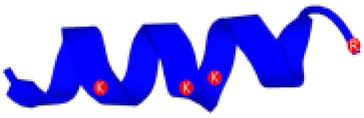 |

| | | | | |
|---|---|---|---|---|
| 25 | Shark (C. milii) | GrpE | 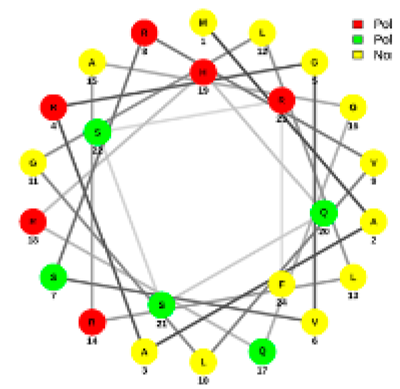 | 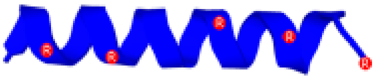 |

**Figure S2.** Flow cytometric analysis of MCCC1-MTS and DBT-MTS cytotoxic activity against HaCaT cells.

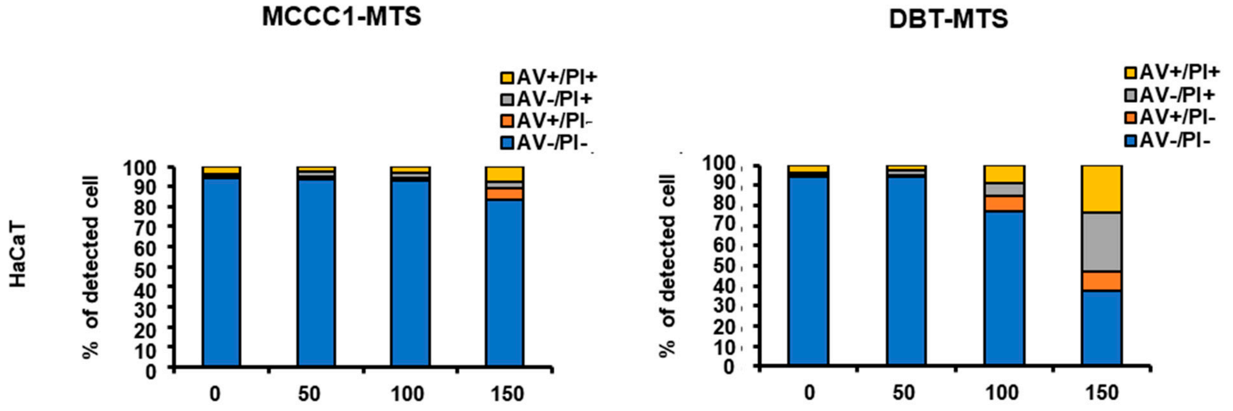

**Figure S3.** Summary of antimicrobial activity spectra of the 25 selected MTSs against E. coli, S. sureus, MRSA, and C. albicans

| MTS ID | *E. coli* | *S. aureus* | MRSA | *C. albicans* |
|---|---|---|---|---|
| Carb_E2C01 | - | - | - | +++ |
| Carb_CLPP | ++ | ++ | - | +++ |
| Carb_Tmlhe | + | ++ | + | +++ |
| Carb_ES1 | + | ++ | - | +++ |
| Carb_TK2 | +++ | +++ | + | +++ |
| Carb_Mmaa | - | - | - | + |
| Carb_Echdc3 | +++ | +++ | - | +++ |
| Carb_COX42 | - | - | - | ++ |
| Carb_Atp5d | - | - | - | ++ |
| Carb_MCCC1 | +++ | +++ | +++ | +++ |
| Carb_Auh | + | + | - | +++ |
| Carb_PMPCA | +++ | ++ | - | +++ |
| Carb_MRPL50 | + | + | - | +++ |
| Carb_Mrpl4 | +++ | +++ | - | +++ |
| Carb_Acot9 | + | + | + | +++ |
| Herring_DBT | +++ | +++ | + | +++ |
| Shrimp_SOD | - | - | - | + |
| Oyster_Sdi1 | - | - | - | + |
| Oyster_MIMP1 | ++ | + | - | ++ |
| BA_TIM44 | + | - | - | ++ |
| B_whale-TIM50 | + | ++ | - | ++ |
| S_whale_Tim29 | + | + | - | +++ |
| Y_whale_Tim29 | + | + | - | +++ |
| Salmon_AIF1 | +++ | +++ | - | +++ |
| Salmon_GrpE | + | + | - | +++ |

- : No activity, + : Mild activity, ++ : Strong activity, +++ : Very strong activity
